# Putting Co-Exposures on Equal Footing: An Ecological Analysis of Same-Scale Measures of Air Pollution and Social Factors on Cardiovascular Disease in New York City

**DOI:** 10.3390/ijerph16234621

**Published:** 2019-11-21

**Authors:** Jamie L. Humphrey, Colleen E. Reid, Ellen J. Kinnee, Laura D. Kubzansky, Lucy F. Robinson, Jane E. Clougherty

**Affiliations:** 1 Department of Environmental and Occupational Health, Dornsife School of Public Health, Drexel University, Philadelphia, PA 19104, USA; jlh563@drexel.edu; 2 Geography Department, University of Colorado Boulder, Boulder, CO 80309, USA; Colleen.Reid@colorado.edu; 3 University Center for Social and Urban Research, University of Pittsburgh, Pittsburgh, PA 15260, USA; ejk40@pitt.edu; 4 Department of Social and Behavioral Sciences, Harvard T.H. Chan School of Public Health, Boston, MA 02115, USA; lkubzans@hsph.harvard.edu; 5 Department of Epidemiology and Biostatistics, Dornsife School of Public Health, Drexel University, Philadelphia, PA 19104, USA; lfr32@drexel.edu

**Keywords:** spatial scale, social factors, socioeconomic position, urban air pollution, cardiovascular disease (CVD)

## Abstract

Epidemiologic evidence consistently links urban air pollution exposures to health, even after adjustment for potential spatial confounding by socioeconomic position (SEP), given concerns that air pollution sources may be clustered in and around lower-SEP communities. SEP, however, is often measured with less spatial and temporal resolution than are air pollution exposures (i.e., census-tract socio-demographics vs. fine-scale spatio-temporal air pollution models). Although many questions remain regarding the most appropriate, *meaningful* scales for the measurement and evaluation of each type of exposure, we aimed to compare associations for multiple air pollutants and social factors against cardiovascular disease (CVD) event rates, with each exposure measured at equal spatial and temporal resolution. We found that, in multivariable census-tract-level models including both types of exposures, most pollutant–CVD associations were non-significant, while most social factors retained significance. Similarly, the magnitude of association was higher for an IQR-range difference in the social factors than in pollutant concentrations. We found that when offered equal spatial and temporal resolution, CVD was more strongly associated with social factors than with air pollutant exposures in census-tract-level analyses in New York City.

## 1. Introduction

Evidence from environmental epidemiology consistently links urban air pollution to a variety of health risks including increased mortality, cardiovascular disease and respiratory disease [1]. It has become standard practice, in this field, to adjust models for potential confounding by socioeconomic position (SEP), because air pollution is often higher in lower-SEP communities [2,3,4] and because many factors associated with lower SEP (i.e., poverty, lower education, violence, poor diet) may directly impact health.

In epidemiology, exposure misclassification can lead to biased estimates of association that can be towards or away from the null, depending on whether the misclassification is differential (i.e., if the measurement of the exposure does not depend on the ‘true’ exposure), and whether the misclassification is dependent (e.g., if there is an unmeasured variable that influences misclassification in one or more covariates) [5]. Comparing associations with health for two different exposures, measured at different resolutions, may reveal stronger associations for the measured with less error, assuming that the errors are not dependent [6].

In air pollution epidemiology, increasing emphasis has been placed on minimizing misclassification in air pollution exposure estimates, in both time and space [7]. Often, however, less attention is given to possible effects of misclassification in the confounders, including SEP. This is perhaps most notable in the commonplace use of demographic or other ecologic (area-level) administrative variables as SEP indicators (e.g., census tract poverty rate), even where individual measures would be preferable. Although an increasing number of studies have been investigating whether and how SEP, and its component social stressors, may modify air pollution-health relationships [8,9,10,11,12,13]), fewer studies have rigorously compared social stressors as *co-exposures* with air pollution [14,15], to assess their *relative* impacts on health.

Many individual-level studies of air pollution health effects estimate pollution exposures at fine spatio-temporal scales—often residence-specific daily estimates, using regulatory data (e.g., U.S. EPA AQS monitors), or estimates derived from land-use regression (e.g., [16,17,18]), spatial interpolation (e.g., [19,20]), or related methods [21]. In contrast, the SEP indicators used are often, at best, individual-level categorical variables (e.g., income category, education level), and there is often an implicit assumption that these factors do not vary over time, although, in actuality, factors such as income can vary greatly over time, particularly for low-income or precariously-employed individuals [22,23]. More commonly, however, investigators rely on area-based administrative data as proxy indicators for SEP, especially for larger cohorts. These indicators tend to be reported as annual averages but are actually collected even less frequently. For example, the U.S. Census is performed once every ten years, and the American Community Survey (ACS) reports annual, three-year, or five-year running averages, reported at census tract or block group. This vast mismatch in temporal and spatial resolution between air pollution and SEP exposure estimates may lead to problematic, and largely unknown, patterning in residual confounding, misleading comparisons of attributable health effects, and unknown problems in testing effect modification (interactions) among variables measured with different accuracy.

A further conceptual challenge is that air pollution and social factors have different *meaningful* scales of variation—an issue related to the ‘Uncertain Geographic Context Problem’ (UGCoP), which states that researchers often lack knowledge of the “true causally relevant” geographic scale at which a given exposure may influence health or other outcomes [24]. For example, air pollution is known to differ sharply within several hundreds of meters of a major road [4,25]; as such, persons living immediately adjacent to highways may have exposures many times higher than those living only a few hundred meters away. This meaningful scale differs by pollutant and urban structure, however; primary pollutants (e.g., nitrogen oxides (NOx)) decay rapidly near-source, while secondary pollutants produced via chemical reactions can be more spatially homogenous [26]. In contrast, some social factors may vary meaningfully at the neighborhood scale, if the “neighborhood” accurately captures a social and political space that is relatively homogenous (as in the case of census sociodemographic data) or delineates access to shared resources (e.g., schools). Other social factors may exert influence on health at very different scales, however; the impact of a violent event on distress and health may be very different, for example, if the event occurs on one’s block, several blocks away, or on the other side of the neighborhood [22,27]. A further nuance is that neighbors can define and delineate neighborhoods differently [28]; researcher-imposed neighborhood delineations are often driven by data availability, rather than meaningful neighborhood scales [29,30].

In terms of temporality, air pollution can vary greatly across seasons, day-to-day, and within-day with meteorology, changes in source intensity (e.g., rush hours), and photochemistry. Many social factors (e.g., neighborhood poverty rate) may change more slowly, although some social factors, such as urban violence, have distinct seasonal and diurnal patterns. Although some studies in air pollution epidemiology and social epidemiology have explored scales of measurement appropriate to each exposure individually (i.e., different radial buffers in air pollution exposures [16], or neighborhood definitions for social stressor measurement [28]), no studies, to our knowledge, have examined spatial scale of measurement on epidemiological effect estimates for both exposures.

In this empirical paper, we aimed to examine the *relative* contributions of air pollution and social factors on CVD, comparing exposures *measured on the same spatial and temporal scale*. To do so, we estimated multiple air pollutants and social factors (i.e., community SEP and violence) at the same spatial (census tract) and temporal (annual) scale in New York City (NYC), and examined their joint association with age-adjusted rates of cardiovascular disease (CVD) emergency department (ED) visits at NYC hospitals from 2005 to 2011.

## 2. Materials and Methods

### 2.1. Design and Data

This ecologic study included all emergency department (ED) visits for CVD (ICD-9 code: 390–459) in NYC from 2005 to 2011. Data were obtained from the New York State Department of Health Statewide Planning and Research Cooperative System (SPARCS). Cases less than 18 years old or older than 95 were excluded from analysis (~2% of cases), in keeping with most studies in this field, because: (1) CVD in children or the very old is likely confounded with multiple co-morbidities, on which we lacked high-quality information; and (2) this small number of cases were too few to substantially alter results. We applied a multi-step address validation and geocoding process [31] for the remaining 1,113,185 case addresses, which we then assigned to NYC census tracts (n = 2167 in total, using year 2010 census boundaries) using point-in-polygon methods.

We examined relationships between socioeconomic position and chronic air pollution exposures on census tract CVD event rates using two composite socioeconomic indicators (a *Socioeconomic Deprivation Index (SDI)* and the *Index of Concentration at the Extremes for Income and Race/Ethnicity (ICE R&I)*, both detailed below), plus a number of social indicators capturing key aspects of social and socioeconomic susceptibility (i.e., *economic/material deprivation, exposure to crime, and racial/ethnic composition)*. Indicators of spatial variation in chronic air pollution exposures were derived from NYC Community Air Survey (NYCCAS) spatial surfaces, as described below.

### 2.2. Air Pollution

Citywide air pollution data were obtained from the NYC Community Air Survey (NYCCAS), one of the largest studies of intra-urban variation in multiple air pollutants. Spatial saturation monitoring of multiple pollutants was performed year-round at 150 sites across all NYC communities for two years, from December 2008 to November 2010 [32]. Land use regressions were used to model long-term average intra-urban spatial variation in fine particles (PM_2.5_) and elemental constituents, nitrogen dioxide (NO_2_), wintertime sulfur dioxide (SO_2_), and summertime ozone (O_3_) [33]. Details about methods, validation, and results from the NYCCAS exposure models for each pollutant are described in [32] and [33]. The NYCCAS surfaces provided annual-average concentration estimates (season-specific averages for SO_2_ and O_3_) at the centroid of every 100 m grid cell across the city, which, for purposes of this analysis, we averaged by census tract.

### 2.3. Social Factors at the Census Tract Level

#### 2.3.1. Felony Assault and Violent Crime Rates

Data on all violent offenses (including murder, assault, burglary, robbery) within NYC from 2006 to 2017 were obtained from NYC OpenData [34]. Rape was excluded, as these crimes are not geocoded. Data were coded as felony assault or violent crime according to the FBI Uniform Crime Reporting program [35]. Crime data included latitude and longitude, which we spatially joined to census tracts, and summed to obtain the total number of felony assaults and total violent crime events per census tract. Crime rates per 10,000 population were calculated using the census tract residential population, obtained from the ACS 2007 to 2011 5 year estimates [36]. Crime rates were created from the 2009 data, which covers the mid-point of our study period; we confirmed that spatial patterns in crime are extremely consistent in NYC, with census tract annual-average crimes rates correlating at *r* > 0.90 across all data years.

#### 2.3.2. Sociodemographic Data

Data on poverty and race/ethnicity at the census tract level were extracted from the 2007 to 2011 ACS 5 year estimates [36], which covers the mid-point and majority of our 2005–2011 study period. These variables include the proportion of the population: living at or below 200% of the federal poverty level (FPL), non-Hispanic white, non-Hispanic black, and Hispanic.

*Socioeconomic Deprivation Index (SDI):* To examine material deprivation, we created a citywide socioeconomic deprivation index (SDI) using a spatially-stratified principal components analysis (PCA) with 25 SEP indicators from the ACS 2007 to 2011 5 year estimates [36] (see Supplemental Material on SDI Methods). This measure was created following standard PCA processes and the methods we used previously [37]. Briefly, local indicators of spatial autocorrelation (LISA) tests were used to identify spatial strata (areas) that maximized internal and minimized external correlation among the 25 SEP variables. We then ran a citywide PCA to remove redundant variables, followed by a PCA within each borough to identity key local variables. The final SDI included eight SEP variables (median household income, % of residents with less than a high school diploma, % of families with annual income <$35,000, % of renter or owner housing costs in excess of 30% of household income, % of households living at or below 200% of FPL, % of households receiving public assistance, % of households receiving food stamp/SNAP benefit, and % of households with an annual income >$50,000). The first factor of the PCA explained 53% of the total variance. We operationalized SDI as the census-tract level interquartile range (IQR) of standardized scores.

**Figure 1:**
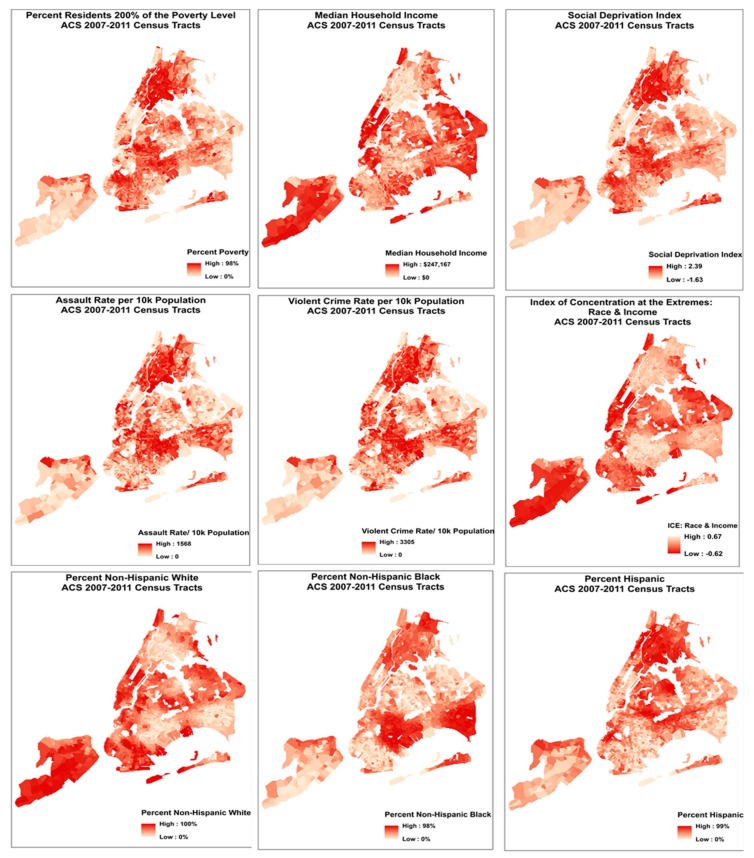
Spatial distribution in social covariates across NYC. Maps are scaled to depict global (overall) patterns across all 2167 census tracts of NYC, corresponding to global analyses presented here, rather than fine-scale differences between individual tracts.

*Index of Concentration at the Extremes: Income + Race/Ethnicity (ICE R&I):* We observed distinct spatial patterning in residential location by race in NYC, which coincided with exposures to chronic social stressors (Figure 1). To begin to explore this racialized economic segregation in NYC, we calculated the Index of Concentration at the Extremes: Income + Race/Ethnicity (ICE R&I) [38,39]. This index is designed to empirically capture entrenched racialized patterns in poverty, wherein low-income black and high-income white persons occupy opposite ends of the U.S. socioeconomic spectrum [39]. This measure quantifies the extent to which an area’s residents are disproportionately comprised of individuals at either extreme of socioeconomic or racial privilege. A value of 1 means that 100% of the population is comprised of white and higher-SEP individuals; a value of −1 indicates that 100% of the population is comprised of black and lower-SEP individuals.

### 2.4. Statistical Analysis

Age-adjusted CVD incidence rates per 100,000 population were calculated for census tracts using the SPARCS ED data and 2000 U.S. Standard Population. Of the 2167 total NYC census tracts, we excluded those with fewer than 200 residents (*N* = 63), leaving *N* = 2104. We further excluded census tracts that were outliers for air pollution and social factors, identified as +/− 3 standard deviations from the mean and, in order to compare across multiple models, performed listwise deletion for missing data across census tracts (*N* = 123). The final sample included *N* = 1981 census tracts. All pollutant and social factor covariates were IQR-standardized.

We quantified correlations among age-adjusted CVD incidence rates, annual-average NO_2_, PM_2.5_, SO_2_, and O_3_, and social factors at the census tract level using Pearson correlation coefficients. We used negative binomial regression to model CVD incidence rates as a function of each air pollutant separately, with and without adjustment for social factors. We first modeled each pollutant and social stressor in separate unadjusted models, then included each social factor in a model simultaneously with each pollutant.

Our social indicators represented three broad categories: (1) *economic/material deprivation* (i.e., SDI, % living at or below 200% of the FPL); (2) *exposure to crime* (i.e., violent crime rate, assault rate), and (3) *racial/ethnic composition* (i.e., % non-Hispanic white, % non-Hispanic black, % Hispanic). In final fully-adjusted models for each pollutant-stressor combination, we adjusted using the strongest predictor from each of the other two stressor categories. (i.e., SDI was the strongest predictor among economic deprivation variables, and thus used as an adjustment variable; violent crime the strongest crime variable; % non-Hispanic black was the strongest ethnicity/race variable.) For example, the final model for NO_2_ and assault rate was adjusted for SDI and % non-Hispanic black, but not for violent crime rate.

Sensitivity analyses were conducted to evaluate the consistency of our estimates. We examined the impacts of spatial autocorrelation on measures of association using Moran’s I and spatial filtering methods to assess and remove spatial autocorrelation from the residuals of negative binomial regression and negative binomial generalized linear models (GLMs). In addition to the census tract-level analyses, we estimated the relative contributions of air pollution and social factors on CVD at the United Hospital Fund area scale (N = 34) (see Materials and Methods UHF in the Supplemental Material). Finally, because CVD is a very broad category, and the mechanisms linking both pollutants and stressors are many and varied, the associations reported here may differ by sub-diagnosis; as a sensitivity-test, we also ran these models for Ischemic Heart Disease (IHD), the most-prevalent sub-diagnosis in our dataset.

Study procedures were reviewed and approved by the Drexel University Institutional Review Board.

## 3. Results

### 3.1. Descriptive Statistics

From January 1, 2005 to December 31, 2011 there were 1,113,185 acute CVD events presented at hospitals in NYC. Table 1 presents summary statistics for age-adjusted CVD incidence rates, average pollutant concentrations, and social factors by census tract. Census-tract CVD incidence rates averaged 14,387 per 100,000 population (range = 1293–58,500). Census-tract mean concentrations of annual-average NO_2_ and PM_2.5_, wintertime SO_2_, and summertime O_3_ concentrations were 24.4 (SD = 3.4), 10.4 (SD = 1.0), 4.4 (SD = 2.0), and 27.2 (SD = 1.5), respectively.

Age-adjusted CVD incidence rates were not strongly correlated with any annual-average air pollutant concentration (Table 2). All social factors were correlated with CVD rates in the hypothesized direction; all were positively correlated with CVD, except for median household income, percent non-Hispanic white residents, and the ICE race and income index, each of which was negatively correlated with CVD rates (Table 2).

As expected, NO_2_, PM_2.5_, and SO_2_ were highly correlated with one another, and negatively correlated with O_3_ (a secondary pollutant). Social factors were not strongly correlated with pollutants, in keeping with our prior results [40].

### 3.2. Unadjusted Models

Table 3 presents estimated unadjusted associations between CVD incidence rates and each annual average pollutant concentration and social factor at the census tract level. In unadjusted models, NO_2_, PM_2.5_, and SO_2_ were significantly positively associated with CVD rates; O_3_ displayed significant protective (negative) associations with CVD rates.

The two social composite indices—the SDI and ICE—were significantly associated with CVD in the hypothesized directions. All other social factors, except median household income and percent non-Hispanic white population, were significantly positively associated with CVD rates. As hypothesized, median household income and percent non-Hispanic white population were negatively associated with CVD rates, such that, for instance, a 1-IQR increase in the percent non-Hispanic white was associated with an expected 35% decrease in CVD incidence rate (IRR = 0.65, 95% CI = 0.64–0.67).

The magnitude of association for each social factor with CVD rates was much higher than for the air pollutants. For example, a 1-IQR increase in NO_2_ was associated with a 9% increase in CVD cases per 100,000 population (IRR = 1.09, 95% CI = 1.07–1.12); in contrast, a one-IQR increase in the SDI was associated with a 43% increase in CVD cases (IRR = 1.43, 95% CI=1.40–1.46), an increase almost 5 times greater.

### 3.3. Mutually-adjusted Models

In mutually-adjusted models (Table 4), examining each pollutant-social factor combination against census-tract CVD rate, the social factors consistently demonstrated a substantial parameter estimate in the hypothesized direction; all were positively associated with CVD, except for the ICE Index, median household income, and percent non-Hispanic white population, all negatively associated with CVD. When adjusted for multiple testing (i.e., false discover rate), some of these associations were no longer significant (shown as not bolded in Table 4).

In most cases, after adjusting for SDI, poverty, or violence rates, air pollution–CVD associations became null. NO_2_ and PM_2.5_ retained statistical significance with adjustment for median household income, the ICE index, and race/ethnic composition. In a few cases, SO_2_–CVD associations reversed direction with adjustment for SDI or poverty. All associations between O_3_ and CVD rate were null, except for an adverse association where adjusting for race/ethnic composition and the ICE index.

After mutually adjusting for each pollutant-social factor combination, the magnitude of association for social factors with CVD incidence rate remained much higher than that of air pollutants. In fact, the IRR for a one-IQR increase in SDI remained at 1.43 (as observed in the unadjusted models [Table 3]) for models adjusted for NO_2_ (95% CI: 1.40−1.46), PM_2.5_ (95% CI: 1.40−1.47), and O_3_ (95% CI: 1.40−1.46) and increased to 1.46 after adjusting for SO_2_ (95% CI: 1.43−1.49).

### 3.4. Fully-adjusted Models

Table 5 presents the results from the fully-adjusted models for each pollutant–social factor combination (i.e., these models were additionally adjusted for the strongest predictor from each of the *other* two social factor categories—the SDI, percent non-Hispanic black residents, and/or violent crime rate). In most cases in these fully-adjusted models, there was no evidence of a significant association between air pollutants and census tract CVD rates. In all cases except for percent Hispanic, the original social factor maintained a significant association in the hypothesized direction, and IRRs for a 1-IQR change in any social factor were several times larger than those for pollutants. Associations for % Hispanic all became null.

All associations between NO_2_ and CVD rate were null, except for a significant protective association in models adjusted for percent non-Hispanic white or Hispanic.

Associations between PM_2.5_ and CVD rate were non-significant, except for significant adverse associations in models adjusted for the ICE index or median household income, and similar to NO_2_, protective associations in models testing percent non-Hispanic white or Hispanic.

Associations between SO_2_ and CVD rate were protective except for null associations in models including percent the ICE index or median household income. All associations between O_3_ and CVD rate were null, except when adjusted for percent Hispanic in which a borderline increased risk was observed.

Compared to the mutually-adjusted models (Table 4), associations between social factors and CVD rate were attenuated in these fully-adjusted models.

### 3.5. Sensitivity Analyses

Most models presented had significant Moran’s *I* values in the residuals, indicating significant spatial autocorrelation in census tract CVD rates *not* accounted for by the pollutants and social variables tested here. In sensitivity analyses (Tables S1–S4), we found that implementing the Moran eigenvector filtering function in negative binomial GLM models provided slightly better model fit and reduced spatial autocorrelation, but associations among air pollutants, social factors, and CVD rates did not differ substantially from negative binomial regression models.

We repeated analyses at the larger United Health Fund (UHF) spatial scale (*n* = 34 in NYC), where a larger suite of social variables was available, supporting factor analyses and identification of spatially-correlated suites of social stressors (see Supplementary Materials about UHF methods) [39]. Results mirror those of the census tract-level analyses; adjusting each air pollutant for each social factor, most pollution-CVD associations became null. Most associations between social factors and CVD rate remained evident after adjusting for any pollutant, and the magnitude of association for each social factor-CVD rate relationship was higher than for air pollutants (Tables S5–S9).

Finally, we considered differences by sub-diagnosis; repeating models with NO_2_ and Ischemic Heart Disease (IHD), we found that IRRs were nearly identical both for NO_2_ and the social factors, using either total CVD or IHD as the outcome (Table S10).

## 4. Discussion

We compared associations with census-tract CVD rates for annual-average concentrations of multiple air pollutants and a range of social factors. We found that, after accounting for social factors, most pollutant-CVD associations were quite weak; in contrast, most social factors retained their association, and the magnitude of association was much higher for a 1-IQR difference in social factors than in pollutant concentrations.

Although substantial questions remain regarding the most appropriate, *meaningful* scales for the measurement and evaluation of air pollutants and social factors in terms of their associations with various health outcomes, our results indicate that, when offered equal spatial and temporal resolution, associations between social factors and CVD are much stronger than are pollutant–CVD associations. It is well known that associations between variables can vary depending on the spatial scale of the analysis (i.e., modifiable areal unit problem (MAUP)) [41], although the true spatial scale of variation for associations between exposures and health outcomes is often unknown (i.e., Uncertain Geographic Context Problem (UGCoP)), and can vary for exposures of very different types (e.g., social stressors and pollution exposures) [24], or for different disease endpoints. Though these exposures may not vary most meaningfully at the census tract level, we used that scale here as a *common* scale of variation to attempt to address the differential spatial misclassification which has often been ignored in epidemiologic studies combining social and environmental exposures. Future research aimed towards identifying the meaningful scale at which environmental and social exposures may influence health may also be improved by focusing on more specific sub-diagnoses, and selecting potential spatial scales of measurement informed by the social and structural properties of the places under study, physical and chemical processes under which pollutants are dispersed and concentrations decay, and the pathophysiology though which specific pollutants or stressors are hypothesized to impact a specific disease under study.

Many studies that investigate the role of air pollutants and measures of social factors on a given health outcome only investigate effect modification of the air pollutant-health outcome by the social factor(s) [42,43], or assess social and environmental exposure in separate models [44]. Our results are in keeping with findings from other health studies investigating co-exposure to environmental and social variables on the same spatial scale. Servadio et al. (2019) assessed the relationship of air pollutants and social factors with respiratory disease and CVD prevalence in Atlanta; with all exposures at the census tract level and adjusting for spatial autocorrelation, they likewise found that, after adjusting for social variables, associations between air pollutants and health were no longer evident, suggesting a larger role for social variables than air pollution in these health outcomes [45]. Similarly, Pala et al. (2019) examined associations between PM_2.5_ and social factors with asthma hospitalizations and ED visits in NYC [46]. They concluded that air pollution was likely not the only determinant of asthma exacerbations, because associations with asthma were weaker for pollutants than for social factors at the UHF42 level. This study did not, however, examine models that included both PM_2.5_ and social factors simultaneously [46]. We observed protective associations between ozone and CVD, as has been noted elsewhere [47,48]. In some locations, this effect has been shown in settings where higher concentrations of ozone, a secondary pollutant, are observed in less-dense, wealthier parts of the study area [49,50], and hence protective effects may be attributable to residual confounding by SEP. In NYC, however, the wealthiest communities in dense areas have substantial primary pollution (i.e., Upper East Side), and consequently lower concentrations of ozone, due to scavenging. Perhaps more importantly, strong chemical scavenging of ozone by fresh emissions leads to an inverse spatial and temporal relationship between NOx and ozone, so much so that NO_2_ (with a negative coefficient) is the strongest spatial predictor of ozone concentrations (*r* = −93) in NYC [51]. As a result, an apparently protective effect of ozone may, in fact, indicate a detrimental impact of fresh combustion emissions on CVD.

### 4.1. Strengths

Our study had a number of notable strengths. First, we have a large sample size (over 1.1 million), and complete hospitals records covering the entire city over several years. Our exposure data includes very fine-scale data on multiple pollutants (100 m resolution), and citywide coverage in a number of key social variables drawn from extensive prior work examining spatial correlations among a wider array of social stressors than in most studies [40], and which has also validated their relationship with resident perceptions of community stressor exposures [52].

Because census tracts are very small in many parts of NYC (*n* = 2167), we were able to examine relations among pollutions, social factors, and health with excellent spatial resolution. We were also able to test relationships at multiple spatial scales (i.e., United Health Fund (UHF) area), in sensitivity analyses, to establish consistency in results.

A tremendous advantage in examining combined effects of pollution and social characteristics in NYC is that NYC has many high-income neighborhoods in dense areas with high pollution concentrations, and vice versa. These “off-diagonal” communities help to ameliorate the challenges associated with persistent spatial confounding between pollution and social stressors in many settings, allowing us to examine independent and interaction effects with lesser risk of persistent confounding and/or off-support inference.

Our primary goal in this paper is to raise the issue of spatial and temporal resolution of co-exposures in epidemiology, and to encourage epidemiologists give more rigorous consideration to the scale of resolution for all exposures/covariates, not only for the main exposure of interest; we believe more attention to scale of resolution will facilitate more accurate estimation of health effects, and a better understanding of the spatial and temporal scales at which very different types of exposures may matter for health. Clearer articulation of what is a “meaningful” spatial scale for various exposures could also help to more effectively target interventions to improve health and to more accurately and characterize susceptible sub-populations and communities where emissions reductions could have greater benefits.

### 4.2. Limitations

Our study also had several limitations. For example, in this analysis we were not able to test associations across multiple time scales. By using annual-average air pollution concentrations, rather than daily concentrations, our study emphasizes relationships between chronic exposures and CVD, rather than examining relationships between acute high pollution exposures and CVD events. Our purpose, however, was to put social and environmental exposures on the same scale. In actuality, crime and other social exposures vary day-to-day and by season; this variation is consistently lost in that most social/sociodemographic data are only available as annual averages, at best (NYC crime rates are reported as 2 year averages. ACS national sociodemographic data are compiled into multi-year estimates). Here, we were required to average pollution measures to one year, to match the previously-aggregated social data. Future studies will use a case-crossover design and related methods to examine individual-level relationships between spatio-temporal pollutant exposure estimates and likelihood of CVD event, with and without effect modification by community-level social covariates.

Although the list of census tract-level social variables we were able to examine here is reasonably long and well-curated, it captures only a limited portion of the depth and complexity of social and sociodemographic patterning across NYC. Future studies will require deeper investigation into patterns of persistent racial residential segregation in NYC, as in other U.S. cities, and related processes that may lead to entrenched spatial clustering in social and economic disadvantage by individual race. Future studies should additionally investigate if there could be other exposures of interest or confounders of the association between air pollution and social stressors on CVD.

The results reported here—particularly those related to higher pollution exposures in higher-SEP communities—may be somewhat unique to NYC [53], although similar relationships have been noted in other major cities (e.g., Rome [54]). Further research should be done to analyze the relative strength of associations between air pollution and social factors on other health outcomes and in other geographic contexts. In addition, studies in other settings should aim to quantify misclassification, and resultant impacts on epidemiological effect estimates and confounding, attributable to mis-characterization of spatial units, or due to comparing multiple exposures measured on very different scales.

We excluded a small number of tracts (n = 63) within the urban area with small populations (<200), generally at the edges of large parks or waterways, due to concerns about (1) the stability of CVD rates in small-denominator tracts, and (2) spatial errors induced by applying rates based on a small population at the edge of a tract across the whole tract (i.e., applying an unstable CVD rate based on a small population at the edge of Central Park, applying that estimate across the whole of a tract that crosses the park). We believe we likely minimized overall error by this exclusion, but this selection bias may somewhat limit the generalizability of our result. Such issues of selection bias and generalizability in spatial data should be considered carefully in subsequent analyses in this and other settings.

Individuals do not spend all of their time in their home census tract; residence-based exposure estimates, however, continue to explain significant variation in CVD and other health outcomes, in part because the residential location represents a small but consistent portion of the individual’s total daily exposure profile, and captures a meaningful portion of the exposure contrast across the cohort. In using the residential location to estimate both pollution and social stressor exposures, we have some degree of misclassification in both, though no reason to believe that the misclassification attributable to using the residential address is necessarily greater for one exposure than the other, which is the comparison of interest for this analysis.

As an ecologic analysis, our study represents a valid comparison across areas (tracts), but observed associations should not be interpreted to be valid at the individual level. Issues of segregation are particularly salient in this regard, as segregation is a process in which individuals are “assigned” to communities (ecologic) based on individual characteristics (i.e., race, wealth). The consequent health effects of segregation are thus based in cross-level (individual-community) interactions, which are beyond the scope of this analysis, but will be addressed in subsequent studies. Finally, reliance on hospital data carries inherent errors related to coding and transcription errors; future studies of CVD and sub-diagnoses may benefit from chart review and other outcomes validation procedures.

## 5. Conclusions

We compared associations with census-tract CVD rates in NYC for annual-average concentrations of multiple air pollutants and a range of social factors. We found that, after accounting for social factors, most air pollutant–CVD associations were no longer evident; in contrast, most social factors retained significance, and the magnitude of association was much higher for an IQR-range difference in social factors than in pollutant concentrations. Future studies of social and environmental exposures on health should carefully consider the relative spatial and temporal resolution of exposure measurement, to account for the influence of both types of exposures on health, to the furthest extent possible.

## Figures and Tables

**Table 1:** Summary statistics by census tract.

	*N*	Min	Mean	Max	Std Dev	Median	IQR
Age-Adjusted CVD/100,000	1981	1293	14,387	58,500	6316	13,046	7671
Average NO_2_	1981	12.7	24.4	38.0	3.4	24.4	3.9
Average PM_2.5_	1981	8.5	10.4	14.4	1.0	10.2	1.4
Average SO_2_	1981	1.4	4.4	11.7	2.0	3.8	2.1
Average O_3_	1981	21.1	27.2	32.9	1.5	27.4	1.6
Socioeconomic Deprivation Index (SDI)*	1981	−1.3	0.1	1.9	0.7	0	1.0
ICE Index: Race and Income (ICE R&I)*	1981	−0.6	0	0.6	0.2	0.0	0.3
200% of Federal Poverty Line	1981	0	0.4	0.9	0.2	0.4	0.3
Median Household Income	1981	9662	54,344	136,053	22,903	51,786	30,679
Violent Crime Rate	1981	0	44.2	323.3	39.0	33.8	44.3
Assault Rate	1981	0	20.3	175.1	20.4	14.2	23.5
Non-Hispanic white	1981	0	0.3	1.0	0.3	0.2	0.6
Non-Hispanic black	1981	0	0.3	1.0	0.3	0.1	0.4
Hispanic	1981	0	0.3	0.9	0.2	0.2	0.3

CVD = cardiovascular disease, NO_2_ = nitrogen dioxide, PM_2.5_ = fine particulate matter, SO_2_ = sulfur dioxide, O_3_ = ozone, Std Dev = Standard Deviation, IQR = interquartile range * SDI and ICE are composite variables.

**Table 2:** Pearson correlations between census-tract level CVD rates, annual-average air pollutant concentrations, and social factors.

	CVD	NO_2_	PM_2.5_	SO_2_	O_3_	SDI Index	ICE: R&I Index	Poverty	Median Income	Violent Crime	Assault Rate	%Non-Hispanic white	% Non-Hispanic black	% Hispanic
Mean Age-Adjusted CVD/100,000	1.00													
Average NO_2_	0.16	1.00												
Average PM_2.5_	0.21	**0.83**	1.00											
Average SO_2_	0.15	0.57	**0.76**	1.00										
Average O_3_	−0.12	**−0.92**	**−0.80**	−0.57	1.00									
Socioeconomic Deprivation Index (SDI)*	**0.60**	0.24	0.33	0.33	−0.16	1.00								
ICE Index: Race and Income*	**−0.64**	−0.10	−0.12	−0.15	0.04	**−0.70**	1.00							
200% of Federal Poverty Line	0.57	0.29	0.37	0.34	−0.20	**0.95**	**−0.63**	1.00						
Median Household Income	−0.51	−0.19	−0.24	−0.27	0.10	**−0.90**	**0.72**	**−0.86**	1.00					
Violent Crime Rate	0.56	0.28	0.28	0.19	−0.19	0.46	−0.53	0.44	−0.39	1.00				
Assault Rate	0.57	0.25	0.26	0.18	−0.17	0.51	−0.56	0.48	−0.43	**0.89**	1.00			
Non-Hispanic white	−0.51	−0.12	−0.15	−0.19	0.06	−0.54	**0.84**	−0.48	0.49	−0.49	−0.50	1.00		
Non-Hispanic black	0.47	−0.02	−0.07	−0.04	0.02	0.17	−0.72	0.10	−0.18	0.40	0.42	**−0.65**	1.00	
Hispanic	0.34	0.18	0.37	0.43	−0.11	0.59	−0.31	0.57	−0.48	0.28	0.30	−0.46	−0.18	1.00

Bolded values indicate stronger correlations, with absolute value >0.60. ICE: R&I = index of concentration at the extremes: race and income; SDI = social deprivation index. *SDI and ICE are composite variables.

**Table 3:** Associations from unadjusted negative binomial regression models, for each pollutant and social factor separately vs. census tract CVD incidence rate. Incidence rate ratio (IRR) represents the change in the CVD incidence rate that occurs with a 1-IQR change in each covariate. Bolded values indicate statistical significance after adjusting for the false discovery rate. *N* = 1981.

	IRR	(95% CI)	AIC
Air Pollutants			
Average NO_2_	**1.09**	**(1.07, 1.12)**	39,765
Average PM_2.5_	**1.14**	**(1.11, 1.17)**	39,721
Average SO_2_	**1.07**	**(1.05, 1.09)**	39,776
Average O_3_	**0.94**	**(0.92, 0.96)**	39,793
Social Factors			
Socioeconomic Deprivation Index	**1.43**	**(1.40, 1.46)**	38,901
ICE: Race and Income	**0.69**	**(0.68, 0.70)**	38,649
% living below 200% of Federal Poverty Line (FPL)	**1.45**	**(1.42, 1.49)**	38,998
Median Household Income	**0.75**	**(0.73, 0.76)**	39,142
Violent Crime Rate	**1.33**	**(1.31, 1.36)**	39,023
Assault Rate	**1.34**	**(1.32, 1.37)**	38,990
% Non-Hispanic white	**0.65**	**(0.64, 0.67)**	39,086
% Non-Hispanic black	**1.35**	**(1.32, 1.38)**	39,264
% Hispanic	**1.21**	**(1.18, 1.24)**	39,567

IRR = incident rate ratio; 95% CI = 95% confidence interval; ICE: R&I = index of concentration at the extremes: race and income; SDI = social deprivation index. SDI and ICE are composite variables. AIC = Akaike Information Criterion. Bolded values indicate significant associations.

**Table 4:** Mutually-adjusted negative binomial models for each pollutant-social factor combination, vs. census tract CVD incidence rate. Incident rate ratio (IRR) represents the change in CVD incidence rate per 1-IQR change in each covariate. Bolded values indicate statistical significance after adjusting for the false discovery rate. *N* = 1981.

		NO_2_			PM_2.5_			SO_2_			O_3_	
Exposures	IRR	(95% CI)	AIC	IRR	(95% CI)	AIC	IRR	(95% CI)	AIC	IRR	(95% CI)	AIC
Pollutant	1.01	(0.99, 1.03)	38,902	1.00	(0.98, 1.02)	38,903	**0.96**	**(0.95, 0.98)**	38,884	1.00	(0.98, 1.01)	38,903
SDI	**1.43**	**(1.4, 1.46)**		**1.43**	**(1.4, 1.47)**		**1.46**	**(1.43, 1.49)**		**1.43**	**(1.4, 1.46)**	
Pollutant	**1.04**	**(1.03, 1.06)**	38,623	**1.07**	**(1.05, 1.09)**	38,597	1.01	(1, 1.03)	38,647	**0.97**	**(0.96, 0.98)**	38,634
ICE: R&I	**0.69**	**(0.68, 0.7)**		**0.69**	**(0.68, 0.71)**		**0.69**	**(0.68, 0.7)**		**0.69**	**(0.68, 0.7)**	
Pollutant	1.00	(0.98, 1.02)	39,000	0.99	(0.97, 1.01)	38,999	**0.97**	**(0.95, 0.98)**	38,984	1.00	(0.99, 1.02)	38,999
% < 200% FPL	**1.45**	**(1.42, 1.49)**		**1.46**	**(1.42, 1.5)**		**1.48**	**(1.44, 1.52)**		**1.46**	**(1.42, 1.49)**	
Pollutant	**1.03**	**(1.01, 1.05)**	39,133	**1.04**	**(1.02, 1.07)**	39,130	0.99	(0.98, 1.01)	39,144	**0.98**	**(0.96, 0.99)**	39,138
Median Income	**0.75**	**(0.74, 0.77)**		**0.75**	**(0.74, 0.77)**		**0.74**	**(0.73, 0.76)**		**0.75**	**(0.73, 0.76)**	
Pollutant	1.00	(0.98, 1.02)	39,025	**1.03**	**(1.01, 1.06)**	39,016	**1.02**	**(1, 1.03)**	39,020	1.00	(0.98, 1.02)	39,025
Violent Crime	**1.33**	**(1.31, 1.36)**		**1.32**	**(1.29, 1.35)**		**1.33**	**(1.3, 1.35)**		**1.33**	**(1.31, 1.36)**	
Pollutant	1.00	(0.99, 1.02)	38,992	**1.03**	**(1.01, 1.05)**	38,984	1.02	(1, 1.03)	38,988	1.00	(0.98, 1.02)	38,992
Assault Rate	**1.34**	**(1.31, 1.37)**		**1.33**	**(1.3, 1.36)**		**1.33**	**(1.31, 1.36)**		**1.34**	**(1.31, 1.37)**	
Pollutant	**1.04**	**(1.03, 1.06)**	39,065	**1.07**	**(1.05, 1.1)**	39,047	1.01	(1, 1.03)	39,086	**0.97**	**(0.95, 0.99)**	39,075
% non-Hispanic white	**0.66**	**(0.64, 0.68)**		**0.67**	**(0.65, 0.68)**		**0.66**	**(0.64, 0.68)**		**0.66**	**(0.64, 0.68)**	
Pollutant	**1.09**	**(1.07, 1.11)**	39,184	**1.16**	**(1.13, 1.18)**	39,093	**1.08**	**(1.06, 1.1)**	39,185	**0.94**	**(0.93, 0.96)**	39,227
% non-Hispanic black	**1.35**	**(1.32, 1.38)**		**1.36**	**(1.33, 1.39)**		**1.35**	**(1.32, 1.38)**		**1.35**	**(1.32, 1.38)**	
Pollutant	**1.05**	**(1.03, 1.08)**	39,544	**1.06**	**(1.03, 1.09)**	39,552	1.00	(0.98, 1.02)	39,569	**0.96**	**(0.94, 0.98)**	39,554
% Hispanic	**1.20**	**(1.17, 1.23)**		**1.18**	(1.16, 1.21)		**1.21**	**(1.18, 1.24)**		**1.20**	**(1.18, 1.23)**	

IRR = incident rate ratio; 95% CI = 95% confidence interval; ICE: R&I = index of concentration at the extremes: race and income; SDI = social deprivation index. SDI and ICE are composite variables. AIC = Akaike Information Criterion..

**Table 5:** Fully-adjusted negative binomial models for each pollutant-social factor combination vs. census tract CVD rate, additionally adjusted for the strongest predictor of CVD from the *other* two social factor categories (SDI in the economic/material deprivation category, Violent Crime Rate in the exposure to crime category, and/or % non-Hispanic black in the racial/ethnic composition category). Incident rate ratio (IRR) represents the change in CVD incidence rate per 1-IQR change in each covariate. Bolded values indicate statistical significance after adjusting the false discovery rate. *N* = 1981.

		NO_2_			PM_2.5_			SO_2_			O_3_	
Exposures	IRR	(95% CI)	AIC	IRR	(95% CI)	AIC	IRR	(95% CI)	AIC	IRR	(95% CI)	AIC
Pollutant	0.99	(0.98, 1.01)	38,192	1.01	(0.99, 1.02)	38,192	**0.98**	**(0.96, 0.99)**	38,182	1.01	(0.99, 1.02)	38,192
SDI	**1.30**	**(1.27, 1.33)**		**1.30**	**(1.27, 1.32)**		**1.31**	**(1.29, 1.34)**		**1.30**	**(1.27, 1.33)**	
Pollutant	1.01	(0.99, 1.02)	38,406	**1.03**	**(1.02, 1.05)**	38,394	1.00	(0.99, 1.01)	38,407	0.99	(0.98, 1.01)	38,406
ICE: R&I	**0.75**	**(0.74, 0.77)**		**0.75**	**(0.74, 0.77)**		**0.75**	**(0.74, 0.77)**		**0.75**	**(0.74, 0.77)**	
Pollutant	0.99	(0.97, 1)	38,205	1.00	(0.98, 1.02)	38,209	**0.98**	**(0.97, 0.99)**	38,200	1.01	(1, 1.03)	38,205
% <200% FPL	**1.33**	**(1.3, 1.36)**		**1.32**	**(1.29, 1.35)**		**1.34**	**(1.31, 1.37)**		**1.33**	**(1.3, 1.36)**	
Pollutant	1.00	(0.99, 1.02)	38,387	**1.03**	**(1.01, 1.05)**	38,376	1.00	(0.98, 1.01)	38,388	1.00	(0.98, 1.01)	38,388
Median Income	**0.82**	**(0.81, 0.84)**		**0.83**	**(0.81, 0.84)**		**0.82**	**(0.81, 0.84)**		**0.82**	**(0.81, 0.84)**	
Pollutant	0.99	(0.98, 1.01)	38,192	1.01	(0.99, 1.02)	38,192	**0.98**	**(0.96, 0.99)**	38,182	1.01	(0.99, 1.02)	38,192
Violent Crime	**1.12**	**(1.1, 1.14)**		**1.12**	**(1.1, 1.14)**		**1.12**	**(1.1, 1.14)**		**1.12**	**(1.1, 1.14)**	
Pollutant	1.00	(0.98, 1.01)	38,224	1.01	(0.99, 1.03)	38,223	**0.98**	**(0.97, 0.99)**	38,216	1.00	(0.99, 1.02)	38,224
Assault Rate	**1.11**	**(1.09, 1.14)**		**1.11**	**(1.09, 1.13)**		**1.12**	**(1.09, 1.14)**		**1.11**	**(1.09, 1.14)**	
Pollutant	**0.98**	**(0.96, 0.99)**	38,423	**0.98**	**(0.96, 1)**	38,425	**0.96**	**(0.95, 0.97)**	38,399	1.01	(1, 1.03)	38,427
% non-Hispanic white	**0.85**	**(0.83, 0.88)**		**0.85**	**(0.83, 0.88)**		**0.85**	**(0.82, 0.87)**		**0.85**	**(0.83, 0.88)**	
Pollutant	0.99	(0.98, 1.01)	38,192	1.01	(0.99, 1.02)	38,192	**0.98**	**(0.96, 0.99)**	38,182	1.01	(0.99, 1.02)	38,192
% non-Hispanic black	**1.21**	**(1.18, 1.23)**		**1.21**	**(1.18, 1.23)**		**1.20**	**(1.18, 1.23)**		**1.21**	**(1.18, 1.23)**	
Pollutant	**0.97**	**(0.96, 0.99)**	38,530	**0.97**	**(0.95, 0.99)**	38,531	**0.96**	**(0.94, 0.97)**	38,513	**1.02**	**(1, 1.04)**	38,535
% Hispanic	0.99	(0.97, 1.02)		1.00	(0.98, 1.02)		1.01	(0.99, 1.03)		0.99	(0.97, 1.01)	

IRR = incident rate ratio; 95% CI = 95% confidence interval; ICE: R&I = index of concentration at the extremes: race and income; SDI = social deprivation index. SDI and ICE are composite variables. AIC = Akaike Information Criterion.

## Supplementary Materials

The following are available online at https://www.mdpi.com/1660-4601/16/23/4621/s1, SDI Methods, Material and Methods—United Hospital Fund (UHF) area, Table S1. OLS and SAR regression models for NO_2_ and social factors vs. age-adjusted census tract CVD event rates. Moran’s I was used to assess autocorrelation in model residuals. All variables are IQR-standardized, Table S2. OLS and SAR regression models for PM_2.5_ and social factors vs. age-adjusted census tract CVD event rates. Moran’s I was used to assess autocorrelation in model residuals. All variables are IQR-standardized, Table S3. OLS and SAR regression models for SO_2_ and social factors vs. age-adjusted census tract CVD event rates. Moran’s I was used to assess autocorrelation in model residuals. All variables are IQR-standardized, Table S4. OLS and SAR regression models for O_3_ and social factors vs. age-adjusted census tract CVD event rates. Moran’s I was used to assess autocorrelation in model residuals. All variables are IQR-standardized, Table S5. Descriptive statistics for air pollutants and social factors at UHF scale (*N* = 34), Table S6. Pearson correlations of pollutants, social factors, and CVD rates at UHF scale (*N* = 34), Table S7. Separate OLS models for UHF-area average pollutant concentrations and social stressors on age-adjusted CVD rates (not mutually adjusted) (*n* = 34), Table S8. Combined (mutually-adjusted) OLS models for UHF-area average pollutant concentrations and social stressors on age-adjusted CVD rates (n = 34), Table S9. OLS and SAR regression models testing bivariate measures of association between CVD and pollutant or social stressor across UHF-34 neighborhoods. Moran’s I to assess autocorrelation in model residuals. Table S10. Fully-adjusted models for each NO_2_-social factor combination vs. census tract CVD and ischemic heart disease (IHD) rate, adjusted for SDI, Violent Crime Rate, and % non-Hispanic black (except where each is main social factor of interest in model).
