# Supplementary figures and images for: Putting Co-Exposures on Equal Footing: An Ecological Analysis of Same-Scale Measures of Air Pollution and Social Factors on Cardiovascular Disease in New York City
Source: Int J Environ Res Public Health. 2019 Nov 21;16(23):4621. doi: 10.3390/ijerph16234621 (PMC6926874; doi:10.3390/ijerph16234621)

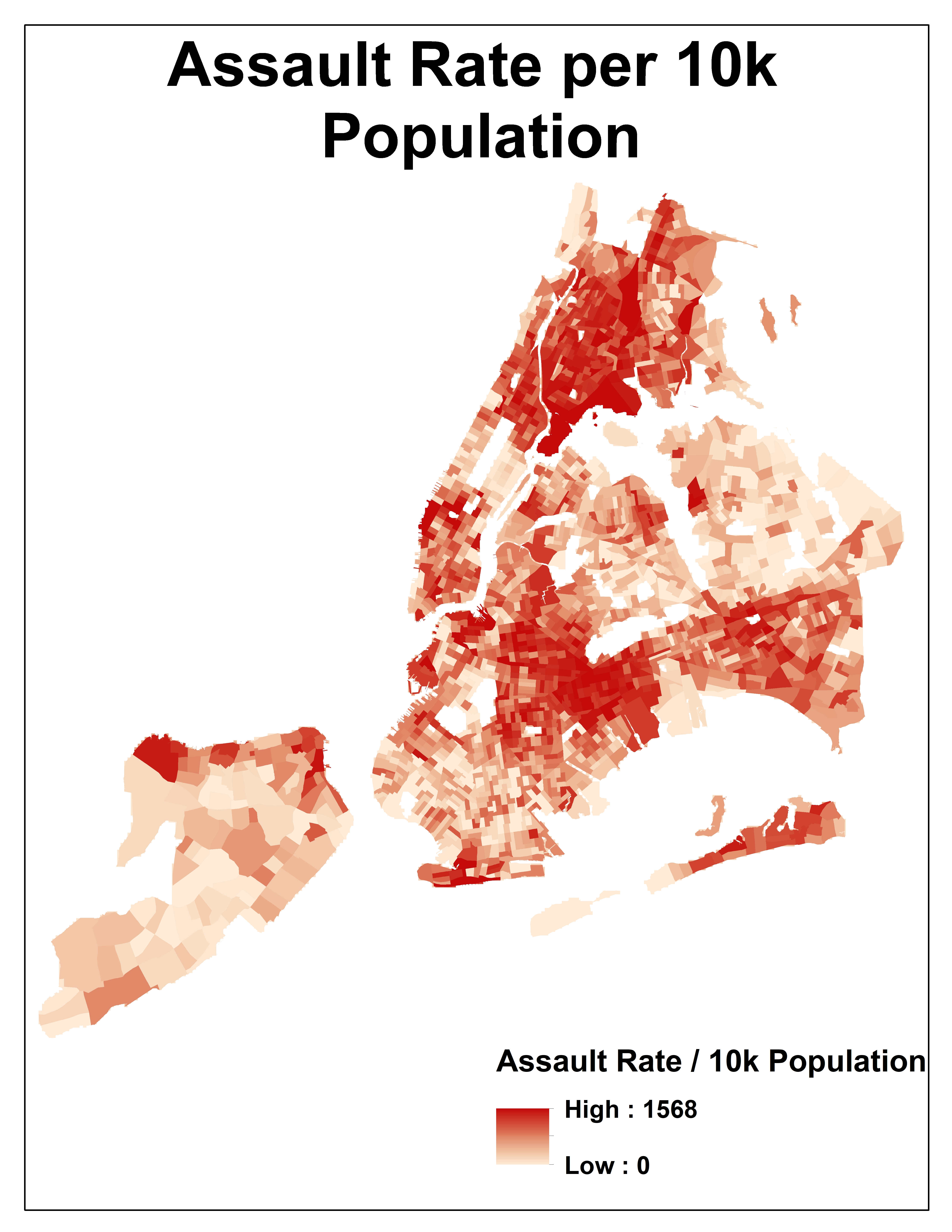

Supplement: Supplementary file 1 [file ijerph-16-04621-s001.zip › ijerph-577604-R2-Supplementary Materials figures/AstRes_WhiteBack.jpg]

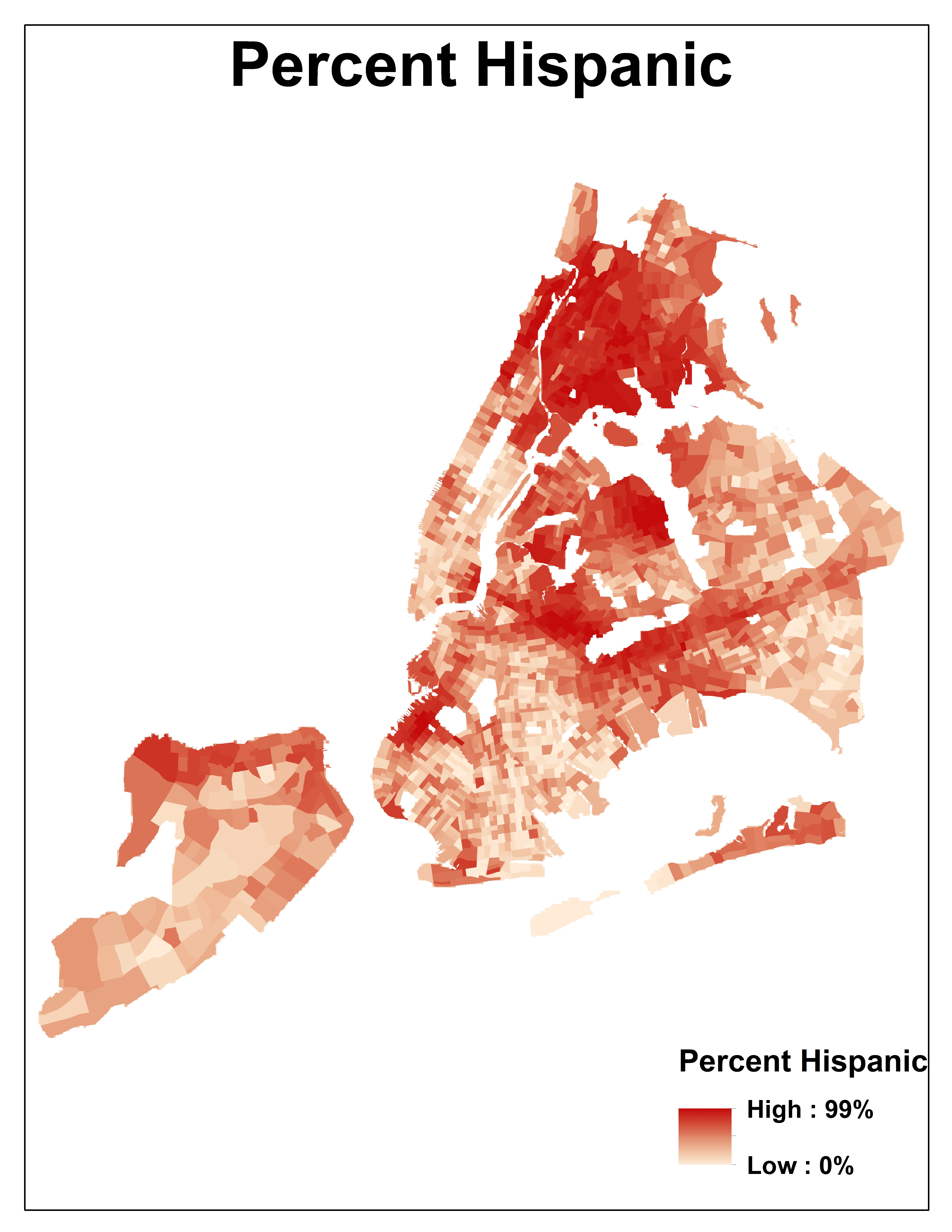

Supplement: Supplementary file 1 [file ijerph-16-04621-s001.zip › ijerph-577604-R2-Supplementary Materials figures/HIsp_WhiteBack.jpg]

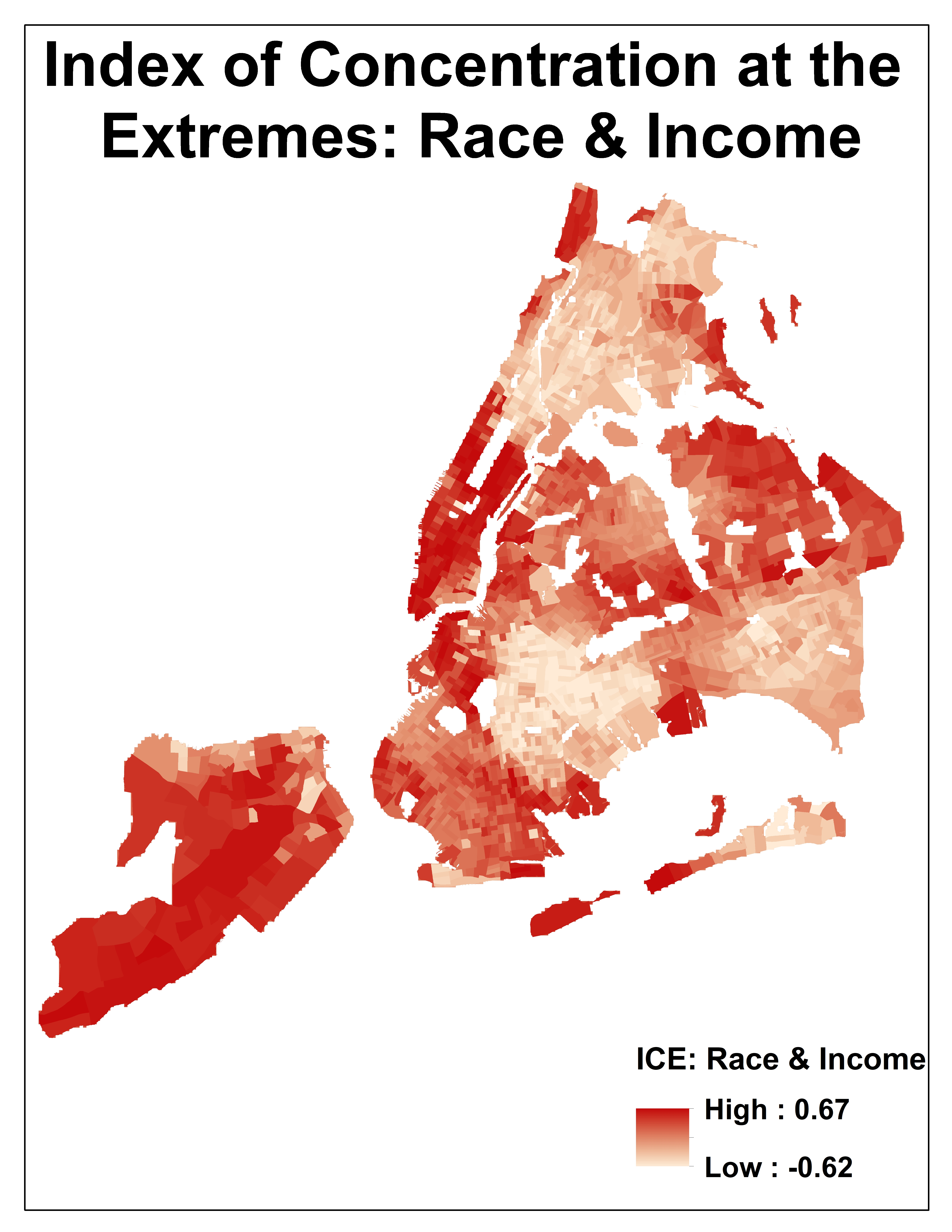

Supplement: Supplementary file 1 [file ijerph-16-04621-s001.zip › ijerph-577604-R2-Supplementary Materials figures/ICE_RI_WhiteBack_FlippedColorRamp.jpg]

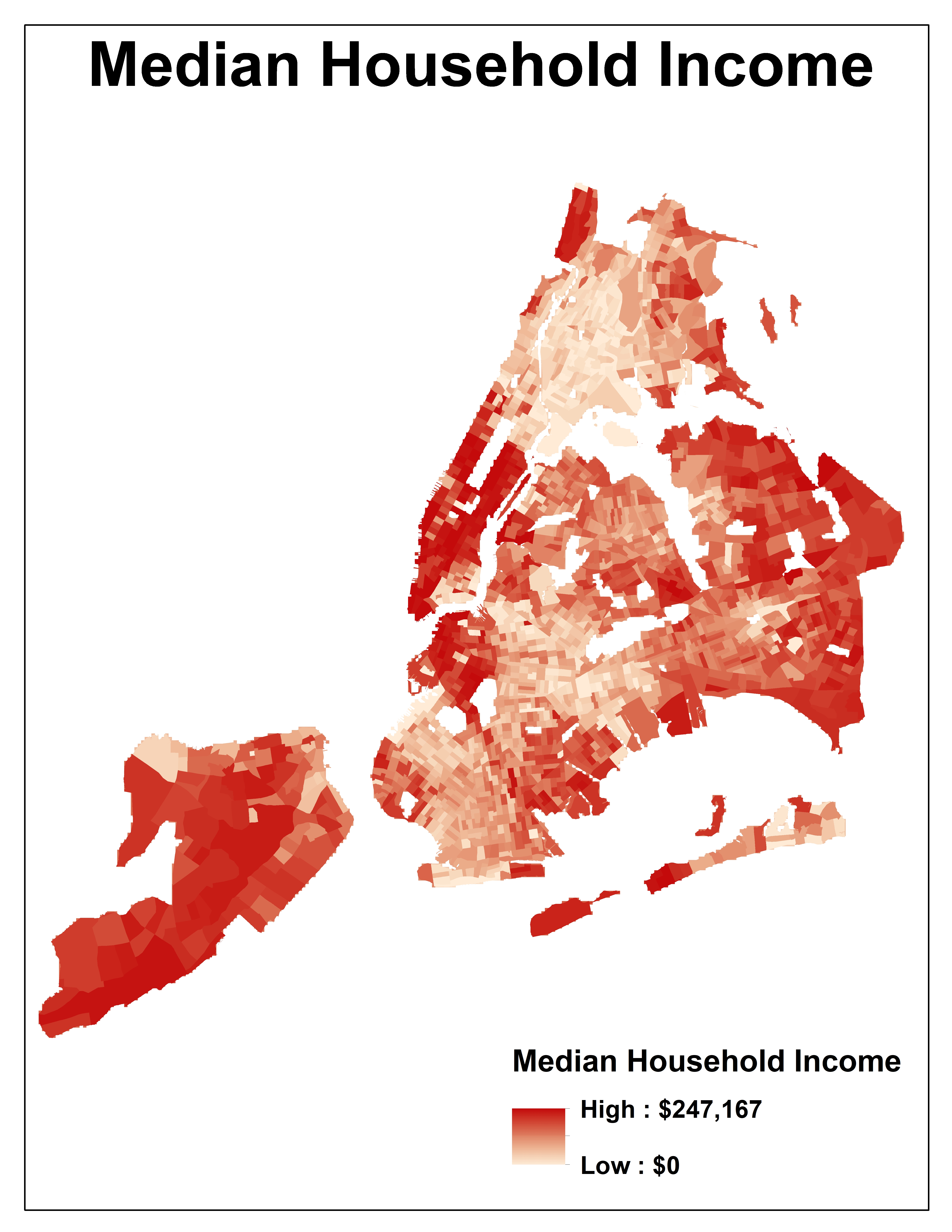

Supplement: Supplementary file 1 [file ijerph-16-04621-s001.zip › ijerph-577604-R2-Supplementary Materials figures/MedHHI_WhiteBack_FlippedColorRamp.jpg]

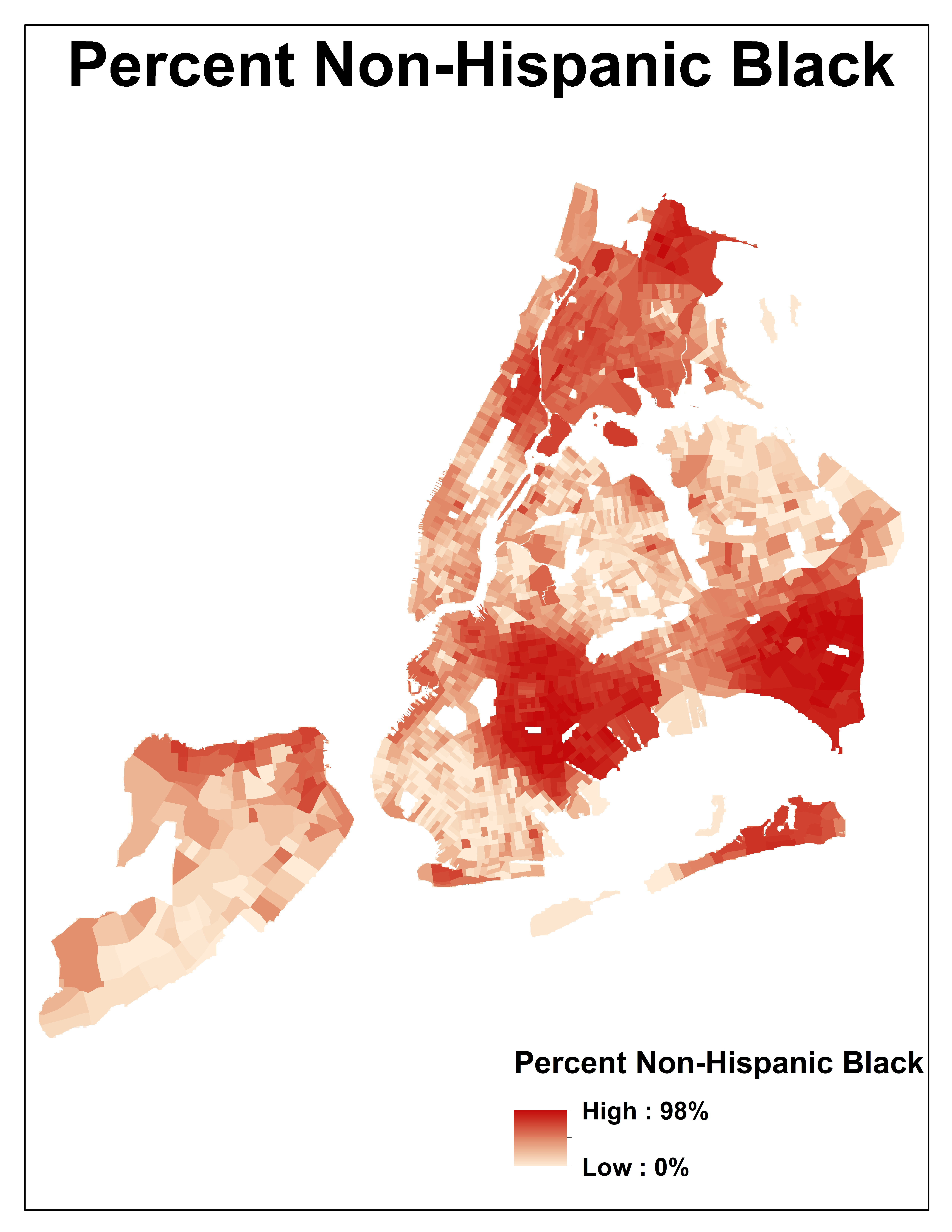

Supplement: Supplementary file 1 [file ijerph-16-04621-s001.zip › ijerph-577604-R2-Supplementary Materials figures/NHB_WhiteBack.jpg]

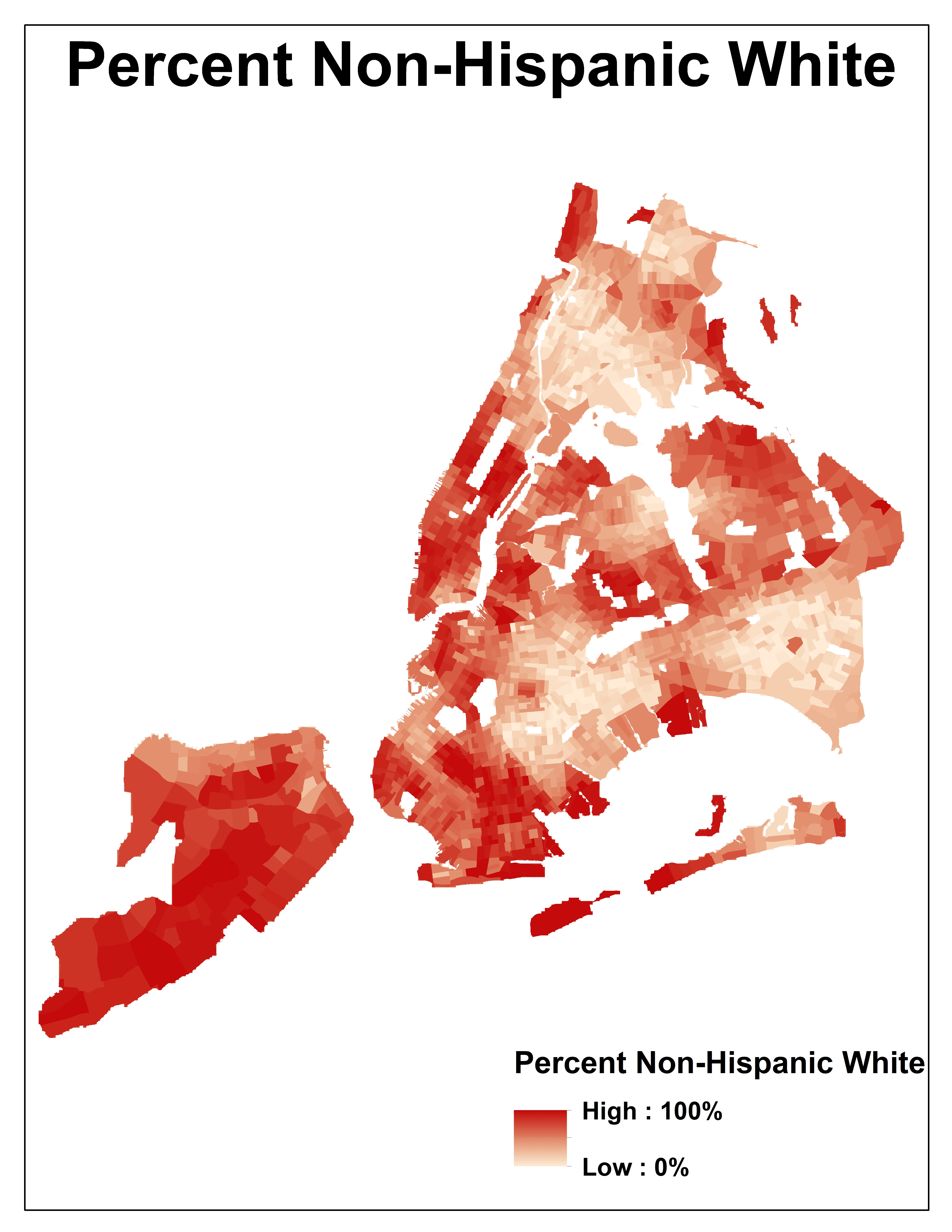

Supplement: Supplementary file 1 [file ijerph-16-04621-s001.zip › ijerph-577604-R2-Supplementary Materials figures/NHW_WhiteBack.jpg]

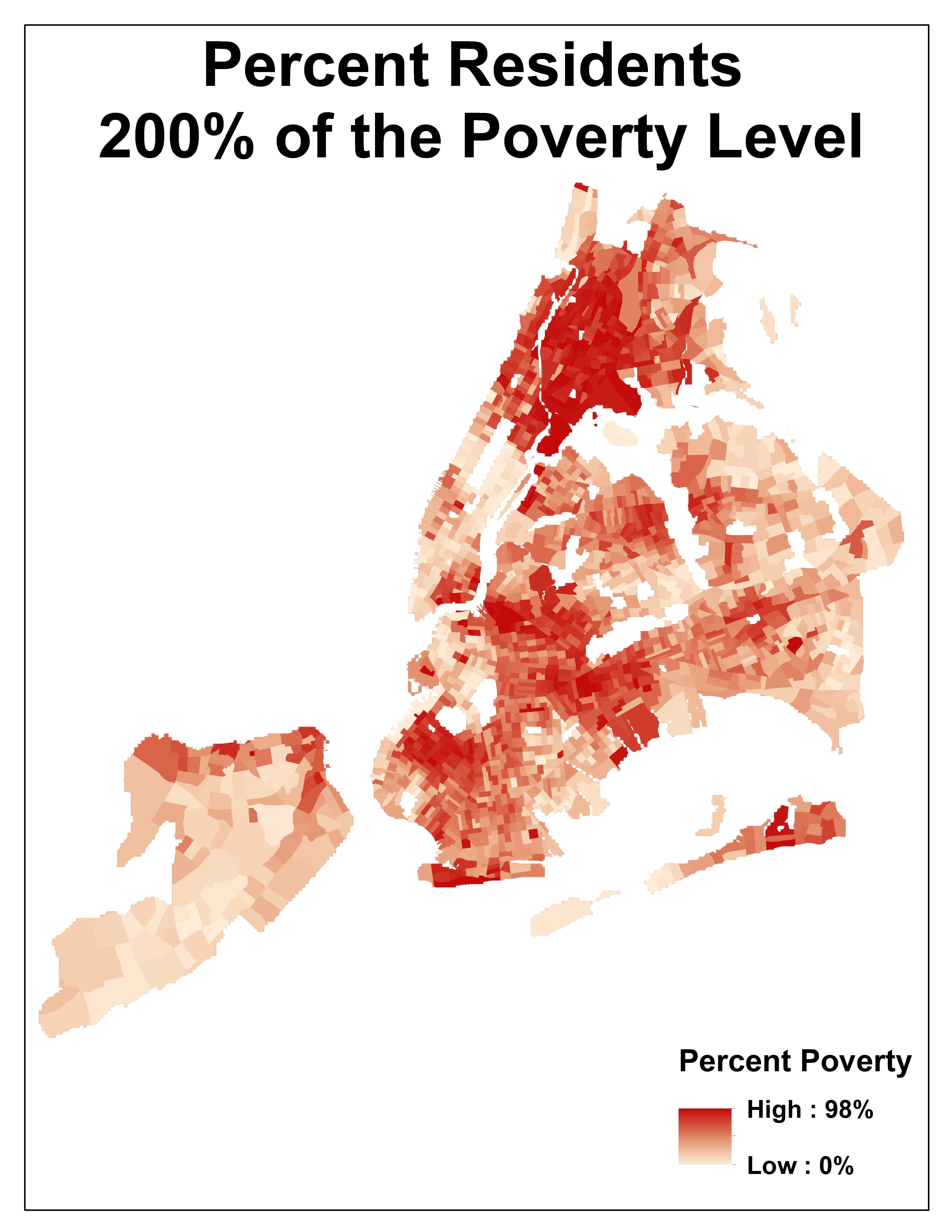

Supplement: Supplementary file 1 [file ijerph-16-04621-s001.zip › ijerph-577604-R2-Supplementary Materials figures/Poverty_WhiteBack.jpg]

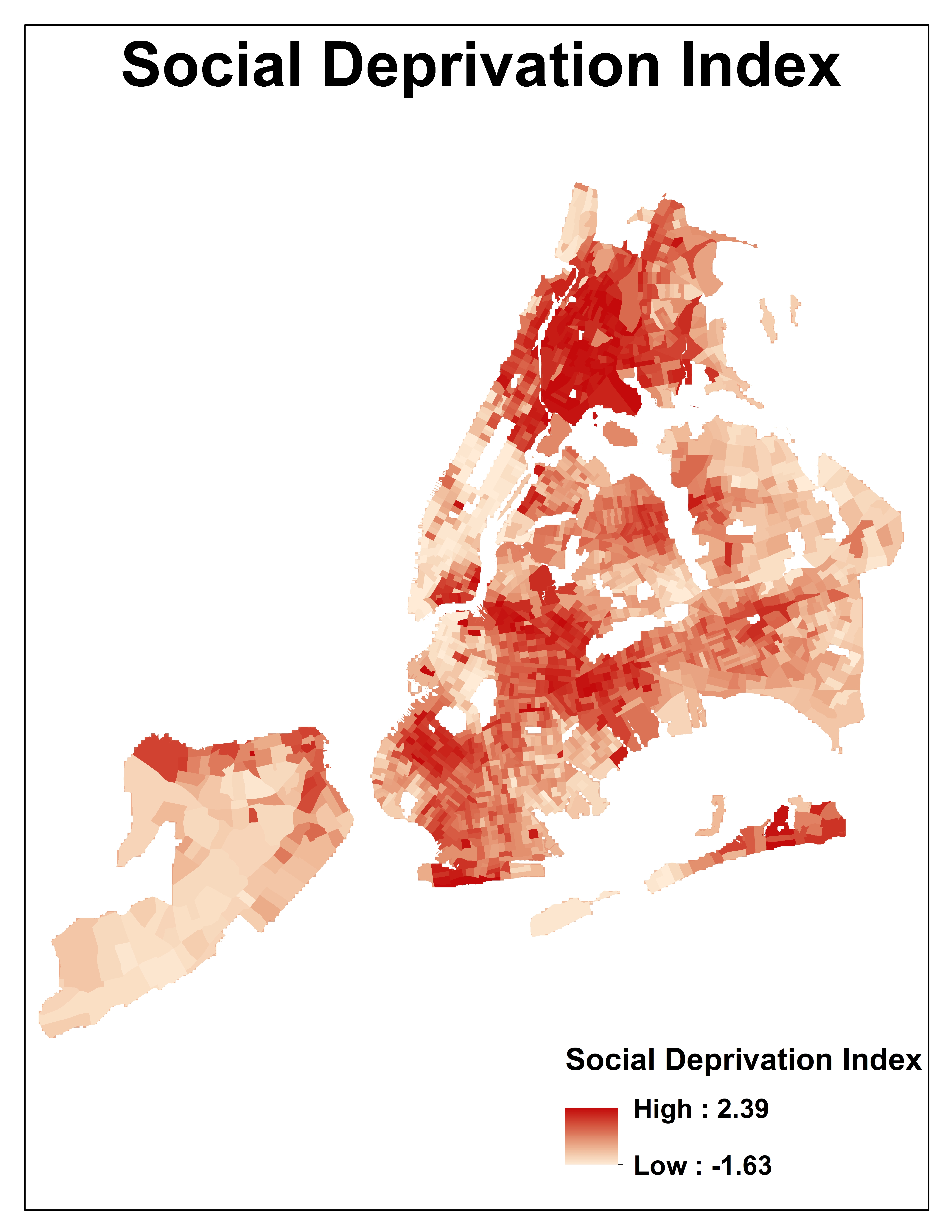

Supplement: Supplementary file 1 [file ijerph-16-04621-s001.zip › ijerph-577604-R2-Supplementary Materials figures/SDI_WhiteBack.jpg]

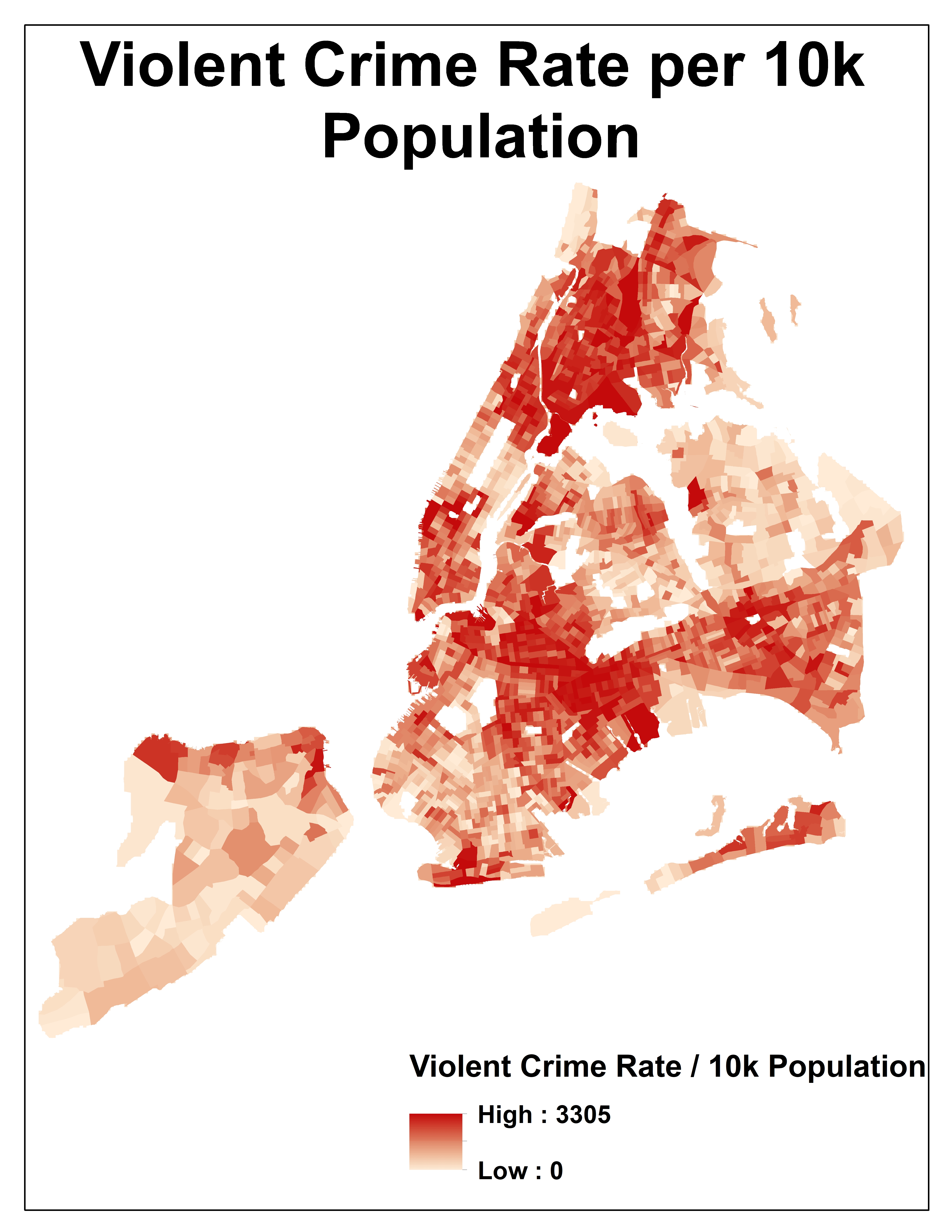

Supplement: Supplementary file 1 [file ijerph-16-04621-s001.zip › ijerph-577604-R2-Supplementary Materials figures/VioRes_WhiteBack.jpg]
